# About lipid metabolism in *Hermetia illucens* (L. 1758): on the origin of fatty acids in prepupae

**DOI:** 10.1038/s41598-020-68784-8

**Published:** 2020-07-17

**Authors:** B. Hoc, M. Genva, M.-L. Fauconnier, G. Lognay, F. Francis, R. Caparros Megido

**Affiliations:** 1 Functional and Evolutionary Entomology, Gembloux Agro-Bio Tech − University of Liège, TERRA, Passage des Déportés 2, 5030 Gembloux, Belgium; 2 Chemistry of Natural Molecules, Gembloux Agro-Bio Tech − University of Liège, TERRA, Passage des Déportés 2, 5030 Gembloux, Belgium

**Keywords:** Zoology, Entomology

## Abstract

Although increasingly targeted in animal nutrition, black soldier fly larvae or prepupae (BSF, *Hermetia illucens* L. 1758) require the characterization and modulation of their fatty acid profile to become fully integrated within the feed sector. This improvement will only be possible by the understanding of underlaying biochemical pathways of fatty acid synthesis in BSF. In this study, we hypothesized a labelling of de novo synthesized fatty acids in BSF by the incorporation of deuterated water (D_2_O) in their feed. Three batches of fifty larvae were reared on two diets with different polyunsaturated fatty acid profiles moistened with 40% of H_2_O or D_2_O: chicken feed or 40% of chicken feed and 60% of flax cake. Although the occurrence of D_2_O in insect feed increased the larval development time and decreased prepupal weight, it was possible to track the biosynthesis of fatty acids through deuterium labelling. Some fatty acids (decanoic, lauric or myristic acid) were exclusively present in their deuterated form while others (palmitic, palmitoleic or oleic acid) were found in two forms (deuterated or not) indicating that BSF can partially produce these fatty acids via biosynthesis pathways and not only by bioaccumulation from the diet. These results suggest the importance of carbohydrates as a source of acetyl-CoA in the constitution of the BSF fatty acid profile but also the potential importance of specific enzymes (e.g.* thioesterase II* or *Δ12 fat2 desaturase*) in BSF fatty acid metabolism. Finally, nearly no deuterated polyunsaturated fatty acids were found in BSF fed with deuterium confirming that BSF is not able to produce these types of fatty acids. Despite the high levels of linolenic acid in flax-enriched diets, BSF will simply bioaccumulate around 13% of this fatty acid and will metabolize approximately two-thirds of it into saturated fatty acids as lauric or myristic acid.

## Introduction

Insects are increasingly identified as an environmentally sustainable source of proteins and lipids for food and feed but also for pharmaceutical or biodiesel applications^1–3^. While yellow mealworm larvae (*Tenebrio molitor *L. 1758) appears to be the preferred model in human nutrition for environmental, nutritional and acceptability considerations^4–6^, larvae or prepupae of black soldier fly (BSF, *Hermetia illucens* L. 1758) emerges by their ability to develop rapidly and extensively on a wide range of decomposing material for the feed sector^7–9^. Although possessing unquestionable nutritional qualities^9,10^, the incorporation of BSF into animal feed is still limited by several factors including their high lipid content (till approximately 40%) and their unbalanced fatty acid profile^10,11^. As BSF fatty acid profile is known to be related with the one of their diet, several experiments have tried to manipulate their fatty acid profile with enriched feed formulation^8,12–14^. By analysing the fatty acid profiles in BSF fed with different diets, it appeared that BSF larvae contain a high level of saturated fatty acids (SFAs, lauric acid C12:0 or myristic acid C14:0) not found in their diet. Moreover, when BSF diets were rich in mono- and polyunsaturated fatty acids (MUFAs, PUFAs), it seemed that BSF larvae bioaccumulate some of them (i.e. oleic acid (*Z*)-C18:1n9; linoleic acid (*Z*,*Z*)-C18:2n6 and α-linolenic acid (*Z*,*Z*,*Z*)-C18:3n3) in order to metabolize them principally into C12:0^8,12–15^. These results suggest that the understanding of biochemical pathways for fatty acid synthesis by BSF will be one of the next challenges for their use as a sustainable source of lipids. Nevertheless, beside the study of Giannetto et al. and Zhu et al. on the expression of key genes involved in BSF lipid metabolism^15,16^, no other studies were performed in order to understand the lipid metabolism in this species of great interest for multiple sectors.

To give insights into BSF fatty acid metabolism, we hypothesized that a complete replacement of water by deuterated water (D_2_O) in diets will allow us to identify which fatty acid is completely/partially biosynthesized by BSF and which one is simply bioaccumulated from their feed. When the only water source for an organism is D_2_O, NADPH, a key coenzyme from the pentose-phosphate cycle, will be produced in deuterated form and lead to deuterium atom incorporation during fatty acid biosynthesis^17–19^. Moreover, in elongation stages, it was shown that deuterium from D_2_O is incorporated into fatty acids by introducing CD_2_ instead of a CH_2_^20,21^. To test this hypothesis, batches of BSF larvae were reared until prepupal stage on PUFAs-differentiated diets (chicken feed or CF and chicken feed with a 60% substitution by flax cake or FL) with water (BSF-CF and BSF-FL) or with deuterated water (BSF-CFD and BSF-FLD with D for deuterated). The fatty acid profile of these larvae was subsequently analysed by gas chromatography coupled with mass spectrometry (GC–MS) in order to identify biosynthesised or bioaccumulated fatty acids.

## Results and discussion

First of all, despite survival rates above 80% on each diet, the presence of D_2_O in insect feed had a negative impact on life history traits of BSF larvae. The individual prepupal weight was significantly higher on standard diets (respectively 251.16 mg ± 4.47a and 200.04 mg ± 6.17b for BSF-CF and BSF-FL; different letters indicate significant differences) than on deuterated diets (respectively 113.56 ± 5.89c and 118.01 ± 6.31c for BSF-CFD and BSF-FLD; F_3,8_ = 134.49; *p* < 0.001) and the larval development was longer on deuterated diets (from 24 days) than on standard diets (from 9 days). By being more stable than H_2_O, D_2_O is effectively known to decrease some biochemical reactions by a special effect called the secondary isotope effect: the more deuterated bonds being found in a compound, the lower will be the compound overall reactivity^22^. Consequently, D_2_O have shown to decrease respiratory cycles or to slow down the heart beat in other Arthropods or Diptera species^22,23^ and may explain growth delays in BSF larvae.

**Figure 1:**
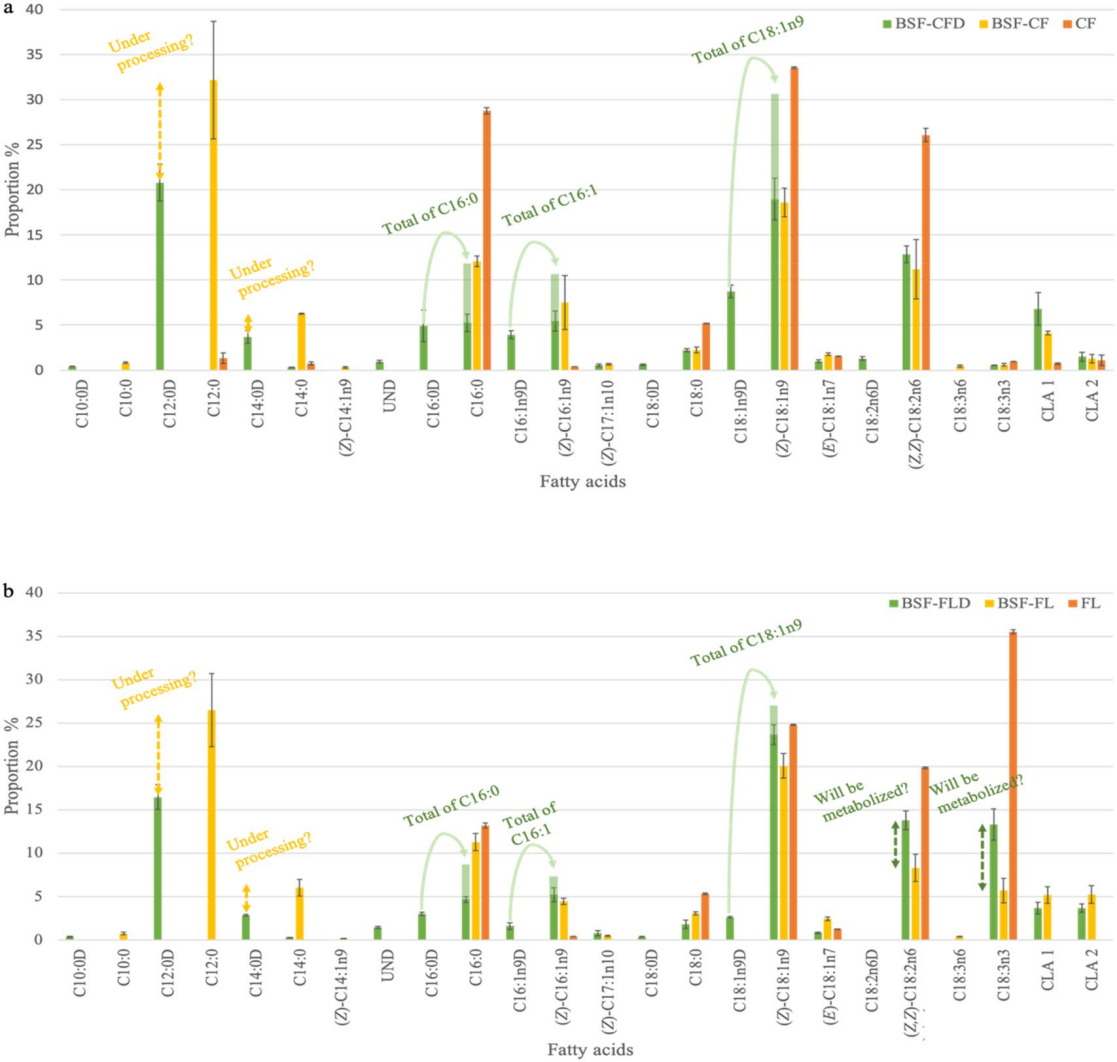
Fatty acid profiles of black soldier fly prepupae fed on differentiated PUFAs diets with H_2_O or D_2_O. (**a**) fatty acid profile of the diet (CF = 100% of chicken feed), of the black soldier fly prepupae produced on this diet with water (BSF-CF) and produced with deuterated water (BSF-CFD). CLA = Conjugated linoleic acid. (**b**) fatty acid profile of the second diet (FL = 40% of chicken feed and 60% of flax cake), of the black soldier fly prepupae produced on this diet with water (BSF-FL) and with deuterated water (BSF-FLD). The total of selected fatty acids represents the sum of the two forms (deuterated or not) of this fatty acid. Since the presence of deuterium has an impact on the kinetics of metabolic reactions, both annotations (*i.e.* “under processing?” and “will be metabolize?”) are related to hypotheses explaining differences in BSF prepupae fatty acid profiles reared on free or containing-deuterium feed. CLA = Conjugated linoleic acid; *Z* and *E* letter describe molecule double bonds stereochemistry.

**Figure 2:**
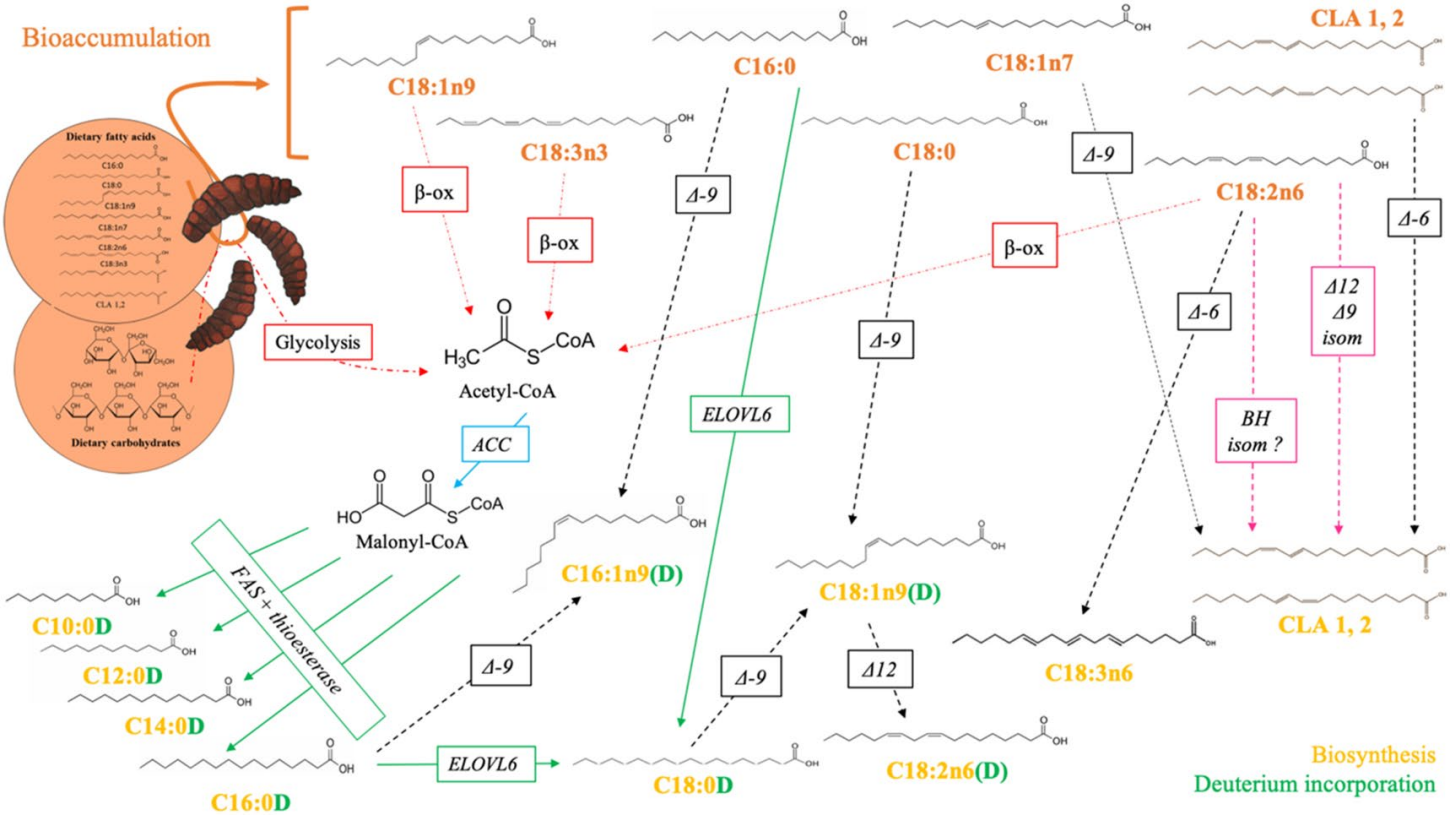
Fatty acid metabolism of black soldier fly prepupae. Whole green arrows = elongation process; whole blue arrow = carboxylation process, red dotted arrows = catabolism process, black dotted arrows = desaturation process, pink dashed arrows = isomerization process. *ACC *Acetyl-CoA carboxylase, *β-ox* β-oxidation, *CLA* Conjugated linoleic acid, *ELOVL *Fatty acid elongase, *FAS *fatty acid synthase, *Δ-X isom* Isomerase, *Δ-X* Desaturase.

The fatty acid profile of BSF-CF showed high level of SFAs (C12:0, C14:0 and C16:0) followed by MUFAs ((*Z*)-C16:1n9 and (*Z*)-C18:1n9) and about fifteen percent of PUFAs (principally (*Z*,*Z*)-C18:2n6) in line with other studies^8,12–15^ (Fig. 1a, Table S1 for details). For BSF-CFD, the global fatty acid profile followed the same pattern as BSF-CF excepted for the appearance of deuterated fatty acids. As expected, decanoic acid (C10:0), lauric acid (C12:0) and myristic acid (C14:0) were almost entirely found in deuterated form as they were not found or in very low proportion (i.e. 0.00% for C10:0, 1.27 ± 0.33% for C12:0 and 0.76 ± 0.18% for C14:0) in the chicken feed. These results confirmed our hypothesis since fatty acids supposed to be fully produced by insects were only present in their deuterated form^17,18^. SFAs from C12:0 to stearic acid (C18:0) are known to be de novo biosynthesized by several insect species from carbohydrates from their diet (Fig. 2). This results was expected for BSF larvae as two genes coding for *acetyl-CoA carboxylase* (*ACC*) and *fatty acid synthase* (*FAS*) were recently characterized^15,24–26^. BSF larvae also produced some proportions of palmitic acid (C16:0) and C18:0. As natural and deuterated C16:0 were found, the present results showed that, despite a high level of this fatty acid in chicken feed (28.78 ± 0.34%), larvae biosynthesized approximately 50% of the C16:0 found in their whole body instead of a simple accumulation. High level in biosynthesized C16:0 could be expected as the fatty acid synthase complex was known to synthetize direct and overwhelming quantity of C16:0 from acetyl-CoA through malonyl-CoA synthesis^27–29^ due to the very high specificity of the terminal thioesterase (which releases the biosynthesized fatty acid) for C16:0^28,30^. Production of C16:0D (or other unexpected deuterated fatty acids) could also result from stress conditions experienced by insects in the presence of D_2_O. Although this hypothesis seems weak based on the expected and obtained results for C:10D and C:12D, combined genomic and proteomic studies, such as the study of Li et al., are essential to improve our knowledge of fatty acid production in BSF^31^. Following this synthesis, C16:0 will be elongated to form longer-chain fatty acids (as C18:0D via the fatty acid elongase 6 /ELOVL 6^32^), desaturated to generate unsaturated fatty acids or used for the synthesis of storage lipids^27^. The occurrence of C10 to C14 fatty acids can be explained by the action of an additional cytosolic thioesterase (e.g.* thioesterase II*) or by retroconversion by partial peroxisomal β-oxidation^28,33,34^. Finally, SFAs’ levels were lower in BSF-CFD than in BSF-CF excepted for the bioaccumulated C18:0. This result can be justified by the decrease in overall reactivity caused by the occurrence of D_2_O in metabolic pathways which reduces the typical production of these fatty acids^22^; the replacement of an hydrogen atom by deuterium has an influence on enzyme activities by reducing their specificities and/or kinetic parameters^20^.

Palmitoleic acid ((*Z*)-C16:1n9) and oleic acid (*Z*)-C18:1n9 are the most represented MUFAs and are both found in natural and deuterated forms. The larger proportion of natural (*Z*)-C18:1n9 within BSF fatty acids revealed a direct bioaccumulation of this molecule from chicken feed lipids. Nevertheless, it was more surprising to identify natural (*Z*)-C16:1n9 since it was nearly absent from the insect diet (0.02 ± 0.00%). This result suggests the potential presence of a *Δ-9-desaturase* gene in BSF larvae coding for a transmembrane fatty acid desaturase using the corresponding saturated homologues to synthesize (*Z*)-C16:1 and (*Z*)-C18:1 as described for *Drosophila melanogaster* Meigen 1830 and *Musca domestica* L. 1758, two other Dipteran species [^35–37^]. During this desaturation step, no hydrogen will be introduced in the fatty acid^38^. Depending on the source of the SFA (deuterated or not), a natural or deuterated MUFA will be produced during the desaturation process and the use of bioaccumulated SFAs (i.e. non-deuterated) to produce MUFAs explain the differences in C16:0 and C18:0 levels between BSF larvae and their diet. The presence of a negligible proportion of C18:0D (produced from C16:0(D) by elongation) could be explained by a quick desaturation of this fatty acid by a *Δ-9-desaturase* to produce the large proportion of C18:1n9D found in this study. The present investigation demonstrated an important total level of C18:1n9 (C18:1n9D + (*Z*)-C18:1n9) in BSF-CFD, significantly higher than in BSF-CF (Table S1; t(4) = −6.82; *p* < *0.001*). Bearing in mind the slower metabolic reaction rates due to D_2_O presence, it can be assumed that part of (*Z*)-C18:1n9 will be β-oxidized into acetyl-CoA to produce C12:0 or C14:0 (see annotation "under processing" for BSF-CFD in Fig. 1a.) as it has previously been demonstrated for *D*. *melanogaster*^36^. Finally, only minute proportions of PUFAs were produced by BSF larvae excepted 1.29 ± 0.22% of deuterated linoleic acid (C18:2n6D). This can be linked to a low activity of a *Δ12 fat2 desaturase* able to convert C18:1n9D into C18:2n6D^39^. Interestingly, it has been shown in *Caenorhabditis elegans* (Maupas, 1900) (Nematoda : Rhabditidae) that the *Δ12 fat2 desaturase* is a bifunctional enzyme that can also catalyse Δ15 desaturation and produce α-linolenic acid ((*Z*,*Z*,*Z*)-C18:3n3) from oleic acid ((*Z*)-C18:1n9) substrate^40^. By studying potential mutations of this gene in BSF, it would be conceivable to consider BSF lines able to produce (*Z*,*Z*,*Z*)-C18:3n3, particularly interesting in animal nutrition^41^. Two other isomers of C18:2 were detected after the (*Z*,*Z*,*Z*)-C18:3n3 peak in chromatograms (Figure S1) and could be identify as conjugated linoleic acids (CLA 1 and 2) as already shown in other studies^42^. The occurrence of CLA remains challenging to explain but leads to various hypotheses. Firstly, as reported elsewhere^1,13^ and shown herein, the presence of (*E*)-vaccenic acid C18:1n7 in low proportion can be linked to the occurrence of CLA by the action of a *Δ9-desaturase*. Nevertheless, this metabolic pathway, typical in ruminants and in humans^43,44^, cannot by itself explain the proportions of CLA found in the present study. Secondly, CLA should also have been produced from microbial biohydrogenation (BH) of PUFAs as it has already been demonstrated in ruminant and suggested by Oonincx et al. for BSF^13,44,45^. BH is divided into three stages: (1) The first reaction is an isomerization that converts C18:2 into different CLA isomers, (2) which are secondarily hydrogenated, leading to the formation of (*E*)-C18:1n7 and finally, (3) a second hydrogenation produces the stearic acid (C18:0)^46^. These hydrogenation step will allow the incorporation of D_2_ into the resulting molecules. Despite the very small proportion of C18:0D, no (*E*)-C18:1n7D was found, indicating that bacteria responsible for BH are unable to perform the second step of the BH (due to specific pH condition?^46^) or are simply absent. As to date, the BSF bacterial community has only been identified at the phylum level, further studies have to be performed to specifically search for BH bacteria (e.g*. Butyrivibrio fibrisolvens* Bryant and Small 1956) as well as the search for strict anaerobiosis condition in the BSF digestive system, essential for bacterial BH^46,47^. A third possible hypothesis to explain CLA occurrence might be the presence of enzymes such as *Δ12* or *Δ9 isomerase* in BSF that allows the isomeric change from linoleic acid ((*Z*,*Z*)-C18:2n6) to CLAs^44,45^. Finally, the appearance of natural γ-linolenic acid ((*Z*,*Z*,*Z*)-C18:3n6) (0.43% ± 0.12) in BSF-CF could be explained by a desaturation of bioaccumulated (*Z*,*Z*)-C18:2n6 by a potential *Δ6 fat3 desaturase*^36,39,48^ while the absence of (*Z*,*Z*,*Z*)-C18:3n6 in BSF-CFD could be explained by a decreased in biochemical reactivity due to the presence of D_2_O.

The second part of this study focused on the incorporation of flax cake (60%) for its (*Z*,*Z*,*Z*)-C18:3n3 supply (Table S1), into chicken feed in order to evaluate any possible modification of BSF fatty acid profile. The BSF-FL(D) showed similar levels of SFAs than BSF-CF(D) despite that the C16:0 proportion in flax cake-based diet was lower than in chicken feed alone. The difference between C16:0 in BSF-FLD and in BSF-CFD could be explained by a lack of acetyl-CoA linked to a lack of carbohydrates in the diet. The incorporation of 60% of flax cake corresponded effectively to a 10% decrease in total carbohydrates in the diet. Moreover, Fig. 1b shows that some PUFAs (linoleic acid (*Z Z*)-C18:2n6 and (*Z*,*Z*,*Z*)-C18:3n3) were in too large proportions in BSF-FLD in comparison with BSF-FL and would have been probably β-oxidized to acetyl-CoA if D_2_O did not delay metabolic reactions (Fig. 1b). The level of produced C12:0 were also reduced in BSF-FL(D) in favour of (*Z*,*Z*,*Z*)-C18:3n3 bioaccumulation into the prepupae in comparison with BSF-CF(D). Concerning MUFAs, C16:1n9D and C18:1n9D were less biosynthesized by BSF-FLD than by BSF-CFD and this reduction could be explained by a decrease in C16:0 availability in diet. Nevertheless, BSF-FLD bioaccumulated higher proportion of (*Z*)-C18:1n9 than BSF-CFD to compensate for this production gap. In both feeds with D_2_O, the total of C18:1n9 (C18:1n9D + (*Z*)*-*C18:1n9) was greater than in H_2_O feeds. The underlying reasons for this excess of total C18:1n9 were unclear but this fatty acid is critical in insects as it is constantly modified depending on physiological requirements^49^. Finally, a PUFA reduction or increase in diet induced a proportional response in the insect: BSF-FL showed lower amounts of (*Z*,*Z*)-C18:2n6 and higher quantities of (*Z*,*Z*,*Z*)-C18:3n3 than BSF-CF. Nevertheless, it seems that, despite high levels of (*Z*,*Z*,*Z*)-C18:3n3 in their diet, BSF prepupae are only able to bioaccumulate around six percent of this fatty acid. In comparison, Oonincx et al*.* have shown that a flax oil incorporation in chicken feed diets could increase (*Z*,*Z*,*Z*)-C18:3n3 levels in BSF larvae up to 9.7%^13^. When looking at the PUFAs’ levels in BSF-FLD (Fig. 1b; Table S1), it could be assumed that PUFAs are properly bioaccumulated by larvae up to almost fifteen percent but are finally metabolized in order to synthetize SFAs (i.e. C12:0, C14:0 and C16:0).

According the results presented herein and thanks to the use of deuterated water as a way of labelling fatty acids, it can be concluded (1) that a strict comparison of fatty acid profiles between the insect and its diet is an oversimplified approach, (2) that fatty acids as palmitic acid (C16:0) or oleic acid ((*Z*)*-*C18:1n9), thought to be merely bioaccumulated from diets were also produced by the BSF itself and (3) that it is mandatory that further studies also focus on the role of carbohydrate level in BSF diets as they are an essential source of acetyl-CoA, a critical molecule in the biosynthesis of fatty acids. Finally, this study also provides the starting point for “omic” investigations of various key enzymes (i.e. *thioesterase II*,* Δ-9-desaturase*, *Δ12 fat2 desaturase* or *Δ6 fat3 desaturase*) involved in fatty acid profiling in BSF. It is also conceivable to only track specific deuterated fatty acids since some of them are available from conventional chemical suppliers and could be incorporated into insect diets. Scientific advances in these different areas will provide a better understanding of the nutritional needs of this insect while exploiting larvae enzymatic natural machinery to modulate their fatty acid profile in order to meet specific nutritional requirements and therefore opens the door to the valorisation of insect supply for the feed industry.

## Material and methods

### Experimental model and subject details

The BSF colonies used for this experiment were reared from an experimental artificial unit of the Functional and Evolutionary Entomology laboratory in Gembloux Agro-Bio Tech (ULiège, Belgium). BSF were produced in a transport container (12.04 × 2.33 × 2.38 m, Jindo, Liaoning, China) converted for mass rearing. The temperature was maintained at 27 ± 1 °C with a relative humidity of 60 ± 5% (data logger; MCH—383 SD, Lutron, Taïwan). Populations of 10 000 individuals were grown in polyvinyl chloride (PVC) magnification tanks (76.50 × 56.50 × 30.50 cm, Auer Packaging, Amerang, Germany) at a density of 2.35 individuals/cm^2^ and fed on brewing byproducts (Spent grain and trub) and carrot peels. Each tank was closed by a lid, a 45° front slope side which ends with an opening connected to a system of self-harvest PVC tube gutter. This rearing facility allowed a self-harvesting of BSF when individuals exit from the feeding substrate during their prepupal stage. Collected prepupae were placed in nylon-cages (75.00 × 75.00 × 115.00 cm, Bugdorm, Taichung, Taiwan) under artificial light with a 6,500 individuals/m^3^ density waiting for imago emergence. When adults emerged, an artificial set up for oviposition was disposed to collect eggs (see Hoc et al., 2019 for more details^50^). The system was placed on a plastic container (17.20 × 11.50 × 6.00 cm, AVA, Temse, Belgium) filled with 7 days old fermented carrot as oviposition attractant and topped by a mosquito net. Eggs were collected daily and incubated for 4 days on an artificial starter diet (laboratory formulation). After one week of growing, young larvae were separated from their substrate by sieving to carry out experiments.

### Feed

Chicken feed (Aveve, Leuven, Belgium) and flax cake mechanically extracted (Scam, Andenne, Belgium) were grinded to 0.750 mm particle size (Pulverisette 19, Fritsch, Germany) for diets formulation. Four different diets were produced based on these two ingredients: 1. 100% of chicken feed with 40% of H_2_O (CF); 2. 40% of chicken feed and 60% of flax cake with 40% of H_2_O (FL); 3. CF with 40% of deuterated water D_2_O (CFD) and 4. FL with 40% of D_2_O (FLD).

### Feeding trial

Experiments were conducted in a controlled dark rearing room (2.45 × 2.06 × 2.72 m) with temperature and relative humidity maintained at 27 ± 1 °C and 60 ± 5%. Three batches of 50 young larvae of 7 days old (0.010 ± 0.002 g) were manually collected and weighed (STX223, Ohaus Scout, Parsippany, USA) for each of the four diets (i.e. CF, FL, CFD, FLD). All batches were reared in plastic containers (108 × 82 × 50 mm) covered with a transparent plastic lid with a square mosquito net (12 × 12 mm) in the centre for ventilation. Containers were randomly arranged at half height on a board in the rearing room. Feed rations were calculated on the basis of 100 mg of fresh diet/larva a day for an estimated developmental period of 15 days (based on previous laboratory data). Consequently, a total of 1.5 g of fresh diet/larva (60% of dry matter) was distributed at the beginning of the experiment and no additional food or water (H_2_O–D_2_O) was added till prepupae collection. The batches were individually fasted for 24 h, washed, dried with a paper towel and were frozen at − 20 °C until analyses.

### Chemical analysis

Diets and prepupae batches (n = 3) have been freeze-dried (Gamma 2–16 LSCPLUS, Martin Christ, Germany) and ground in a blender (IKA A10, Staufen, Germany). Fat content was extracted by the method of Folch (Folch et al. 1957)^51^. Fatty acid composition was determined by GC–MS. Fatty acids of 10.0 mg of lipids were converted into fatty acid methyl esters with boron trifluoride (Sigma-Aldrich, Overijse, Belgium) and methanol (VWR, Oud-Heverlee, Belgium). Fatty acid methyl esters were diluted in 8 mL of hexane (VWR) and analysed with a model 6890n GC System/5973 Mass Selective Detector (Agilent Technologies, Santa Clara, USA), which was fitted with a split/splitless injector (240.0 °C) in splitless mode (splitless time: 0.85 min) and a flame ionization detector (250.0 °C). A Carbowax DA column (Restek Corp., Bellefonte, PA, USA) (30.00 m × 0.25 μm × 0.25 mm in length × thickness × diameter) was used for the analysis. The temperature program was as follows: hold at 55.0 °C for 1 min, increase to 250.0 °C at 10.0 °C/min and hold at 250.0 °C for 5 min. As chromatographic conditions were similar, fatty acid methyl esters were identified based on their retention data compared to a reference mixture of 37 key fatty acid methyl esters (Supelco 37 component FAME mix, Sigma-Aldrich, Overijse, Belgium). Fatty acids were also identified by their retention index and their recorded mass spectra, which were compared with the National Institute of Standards and Technology (NIST) and Wiley spectral databases. The relative percentage of each compound was realized by comparing individual peak area with the sum of peak areas of all identified compounds, using Chemstation software (Agilent Technologies, Palo Alto, CA, USA).

Non-deuterated methyl esters were identified on the basis of their retention index, according to their mass spectrum in comparison with a library and according to their mass fragments. For the example of methyl laurate, the lauric acid (C12:0) methyl ester, whose calculated retention index was 1808, identification of the molecular ion (M+ ; 214 m/z) and GC–MS fragments (M+—C2H5; 185 m/z), (M+—C3H7; 171 m/z), (M+—C4H9; 157 m/z), (M+—C5H11; 143 m/z), (M+—C6H13; 129 m/z), (M+—C7H15; 115 m/z), (M+—C8H17; 101 m/z), allowed the confirmation of the molecule identity (Figure S2a). Ion at 183 m/z corresponded to the loss of OCH3; while ions at 74 and 87 m/z were formed due to McLafferty cleavage and rearrangement^52^. Fatty acids methyl esters were identified on the basis of their retention times, as deuterated molecules were eluted just before their non-deuterated forms^53^ (Figure S1). The identifications were also performed according to their mass spectrum. As an example, for methyl laurate, mass spectrum data for the deuterated sample showed molecular ions between 219 and 222 m/z. As non-deuterated methyl laurate molecular ion is 214 m/z, this showed the incorporation of 5 to 8 deuterium atoms in lauric acid by insect feed with deuterated water in diet. The 221 m/z being the most abundant, the most frequent form of deuterated methyl laurate show an incorporation of 7 deuterium atoms. The analysis of deuterated methyl laurate fragments allowed to determine the position of most deuterium atoms on the acyl chain (Figure S2b). The McLafferty ion at 75 m/z on the mass spectrum, in comparison with 74 m/z for the non-deuterated molecule, confirmed the presence of a deuterium on C11. No deuterium was incorporated on C10 and C9 because the mass difference between ions on deuterated and non-deuterated spectra (88 and 87; 102 and 101; respectively) was one m/z, already due to the deuterium incorporation at C11 position. The difference between ions at 117 and 115 m/z, and at 132 and 129 m/z; on deuterated and non-deuterated spectra, respectively, showed the presence of a deuterium atom at C8 and C7 positions. No deuterium atom was incorporated at C6, as the 3 mass units of difference between ions in deuterated and non-deuterated spectra (146 and 143) was due to deuterium atoms at C11, C8 and C7. Ions at 161 and 157 m/z; and at 176 and 171 m/z on deuterated and non-deuterated spectra, respectively, showed the presence of deuterium atoms at C5 and C4 positions. Additionally, to those determined positions, two supplemental deuterium atoms were situated on the third last carbons, C1, C2 and C1. However, the mass fragments did not allow to determine their precise positions.

### Statistical analysis

All analyses were conducted with RStudio (Version 1.2.1335, Boston, USA) for Mac. The results were presented as the mean and the standard error of the mean (± SE). The accepted level of significance was 5% in all analyses. As data were not normally distributed and/or did not have homogeneous variances, Welch’s T-Test were used to evaluate the influence of deuterium on the fatty acid composition of BSF prepupae reared on CF or on FL.

## Supplementary information

Supplementary information
