# Supplementary material for: About lipid metabolism in Hermetia illucens (L. 1758): on the origin of fatty acids in prepupae
Source: Sci Rep. 2020 Jul 17;10:11916. doi: 10.1038/s41598-020-68784-8 (PMC7368053; doi:10.1038/s41598-020-68784-8)
Supplement: Supplementary file 1 — Supplementary information [file 41598_2020_68784_MOESM1_ESM.docx]

**About lipid metabolism in *Hermetia illucens* (L. 1758). On the origin of fatty acids in prepupae.**

Hoc B. ^1^, Genva M.^2^, Fauconnier M-L.^2^, Lognay G.^1^, Francis F. ^1^ & Caparros Megido R. ^1*^

^1^Functional and Evolutionary Entomology; Gembloux Agro-Bio Tech – University of Liège, TERRA, Passage des Déportés 2, 5030, Gembloux, Belgium.
^2^ Chemistry of Natural Molecules; Gembloux Agro-Bio Tech – University of Liège, TERRA, Passage des Déportés 2, 5030, Gembloux, Belgium.

*Correspondence to: r.caparros@uliege.be

| Table S1. Fatty acid profiles (% of total fatty acids) of experimental diets and black soldier fly prepupae | | | | | | | | | |
| --- | --- | --- | --- | --- | --- | --- | --- | --- | --- |
| ID number | Fatty acid | CF | BSF CFD | BSF CF | **Welch’s T-Test** | FL | BSF FLD | BSF FL | **Welch’s T-Test** |
| 1 | C10:0D | 0.00 ± 0.00 | 0.40 ± 0.04a | 0.00 ± 0.00b | **t**(**4**)**= -22.30**  ***p < 0.001*** | 0.00 ± 0.00 | 0.35 ± 0.06a | 0.00 ± 0.00b | **t**(**4**)**= -10.08**  ***p < 0.001*** |
| 2 | C10:0 | 0.00 ± 0.00 | 0.00 ± 0.00a | 0.82 ± 0.08b | **t**(**4**)**= 16.98**  ***p < 0.001*** | 0.00 ± 0.00 | 0.00 ± 0.00a | 0.75 ± 0.13b | **t**(**4**)**= 10.30**  ***p < 0.001*** |
| / | C10:0D + C10:0 | 0.00 ± 0.00 | 0.40 ± 0.04a | 0.82 ± 0.08b | **t**(**4**)**= 8.39**  ***p < 0.001*** | 0.00 ± 0.00 | 0.35 ± 0.06a | 0.75 ± 0.13b | **t**(**4**)**= 4.97**  ***p = 0.017*** |
| 3 | C12:0D | 0.00 ± 0.00 | 20.81 ± 2.02a | 0.00 ± 0.00b | **t**(**4**)**= -17.88**  ***p < 0.001*** | 0.00 ± 0.00 | 16.45 ± 1.40a | 0.00 ± 0.00b | **t**(**4**)**= -20.39**  ***p < 0.001*** |
| 4 | C12:0 | 1.32 ± 0.57 | 0.00 ± 0.00a | 32.18 ± 6.53b | **t**(**4**)**= 8,53**  ***p < 0.001*** | 0.00 ± 0.00 | 0.00 ± 0.00a | 25.46 ± 4.22b | **t**(**4**)**= 10.86**  ***p = 0.008*** |
| / | C12:0D + C12:0 | 1.32 ± 0.57 | 20.81 ± 2.02a | 32.18 ± 6.53a | **t**(**4**)**= 2,88**  **p = 0.084** | 0.00 ± 0.00 | 16.45 ± 1.40a | 25.46 ± 4.22b | **t**(**4**)**= 3.90**  ***p =0.043*** |
| 5 | C14:0D | 0.00 ± 0.00 | 3.769 ± 0.71a | 0.00 ± 0.00b | **t**(**4**)**= -9.03**  ***p = 0.012*** | 0.00 ± 0.00 | 2.85 ± 0.11a | 0.00 ± 0.00b | **t**(**4**)**= -46.77**  ***p < 0.001*** |
| 6 | C14:0 | 0.76 ± 0.18 | 0.32 ± 0.03a | 6.28 ± 0.06b | **t**(**4**)**= 167.83**  ***p < 0.001*** | 0.00 ± 0.00 | 0.29 ± 0.02a | 6.01 ± 0.95b | **t**(**4**)**= 10.45**  ***p < 0.001*** |
| / | C14:0D + C14:0 | 0.76 ± 0.18 | 4.01 ± 0.71a | 6.28 ± 0.06b | **t**(**4**)**= 5,51**  ***p = 0.031*** | 0.00 ± 0.00 | 3.14 ± 0.10a | 6.01 ± 0.95b | **t**(**4**)**= 5.20**  ***p = 0.033*** |
| 7 | (*Z*)-C14:1n9 | 0.00 ± 0.00 | 0.00 ± 0.00a | 0.30 ± 0.10b | **t**(**4**)**= 4,95**  ***p < 0.038*** | 0.00 ± 0.00 | 0.00 ± 0.00a | 0.19 ± 0.02b | **t**(**4**)**= 16.50**  ***p = 0.003*** |
| 8 | UND | 0.00 ± 0.00 | 0.93 ± 0.13a | 0.00 ± 0.00b | **t**(**4**)**= -12,38**  ***p < 0.001*** | 0.00 ± 0.00 | 1.45 ± 0.10a | 0.00 ± 0.00b | **t**(**4**)**= -25.58**  ***p < 0.001*** |
| 9 | C16:0D | 0.00 ± 0.00 | 4.90 ± 1.75a | 0.00 ± 0.00b | **t**(**4**)**= -4,83**  ***p = 0.041*** | 0.00 ± 0.00 | 3.00 ± 0.16a | 0.00 ± 0.00b | **t**(**4**)**= -32.44**  ***p < 0.001*** |
| 10 | C16:0 | 28.78 ± 0.34 | 5.24 ± 0.98a | 12.07 ± 0.59b | **t**(**4**)**= 10,38**  ***p < 0.001*** | 13.20 ± 0.29 | 4.67 ± 0.32a | 11.28 ± 0.98b | **t**(**4**)**= 11.05**  ***p < 0.001*** |
| / | C16:0D + C16:0 | 28.78 ± 0.34 | 10.14 ± 2.71a | 12.07 ± 0.59a | **t**(**4**)**= 1,21**  **p = 0.344** | 13.20 ± 0.29 | 7.67 ± 0.34a | 11.28 ± 0.98b | **t**(**4**)**= 5.99**  ***p = 0.015*** |
| 11 | C16:1n9D | 0.00 ± 0.00 | 3.93 ± 0.43a | 0.00 ± 0.00b | **t**(**4**)**= -15.97**  ***p < 0.001*** | 0.00 ± 0.00 | 1.60 ± 0.39a | 0.00 ± 0.00b | **t**(**4**)**= -7.17**  ***p = 0.017*** |
| 12 | (*Z*)-C16:1n9 | 0.02 ± 0.00 | 5.42 ± 1.12a | 7.49 ± 3.00a | **t**(**4**)**= 1.12**  **p = 0.357** | 0.02 ± 0.00 | 5.21 ± 0.82a | 4.46 ± 0.35a | **t**(**4**)**= -1.45**  **p = 0.250** |
| / | C16:1n9D + (*Z*)-C16:1n9 | 0.00 ± 0.00 | 9.35 ± 0.71a | 7.49 ± 3.00a | **t**(**4**)**= -1.04**  **p = 0.396** | 0.00 ± 0.00 | 6.81 ± 1.20a | 4.46 ± 0.35a | **t**(**4**)**= -3.25**  **p = 0.067** |
| 13 | (*Z*)-C17:1n10 | 0.00 ± 0.00 | 0.53 ± 0.17a | 0.66 ± 0.08a | **t**(**4**)**= 1.15**  **p = 0.335** | 0.00 ± 0.00 | 0.77 ± 0.31a | 0.47 ± 0.05a | **t**(**4**)**= -1.60**  **p = 0.242** |
| 14 | C18:0d | 0.00 ± 0.00 | 0.59 ± 0.05a | 0.00 ± 0.00b | **t**(**4**)**= -8.44**  ***p < 0.001*** | 0.00 ± 0.00 | 0.37 ± 0.01a | 0.00 ± 0.00b | **t**(**4**)**= -6.87**  ***p < 0.001*** |
| 15 | C18:0 | 5.18 ± 0.04 | 2.21 ± 0.19a | 2.23 ± 0.31a | **t**(**4**)**= 0.09**  **p = 0.933** | 5.33 ± 0.12 | 1.81 ± 0.45a | 3.08 ± 0.19b | **t**(**4**)**= 4.32**  ***p = 0.029*** |
| 16 | C18:1n9D | 0.00 ± 0.00 | 8.72 ± 0.71a | 0.00 ± 0.00b | **t**(**4**)**= -2124**  ***p < 0.001*** | 0.00 ± 0.00 | 2.62 ± 0.07a | 0.00 ± 0.00b | **t**(**4**)**= -69.61**  ***p < 0.001*** |
| 17 | C18:1n9 | 33.55 ± 0.08 | 18.40 ± 2.33a | 18.60 ± 1.59a | **t**(**4**)**= -0.24**  **p = 0.823** | 24.82 ± 0.05 | 23.31 ± 1.14a | 20.07 ± 1.42b | **t**(**4**)**= -3.42**  ***p = 0.028*** |
| / | C18:1n9D + C18:1n9 | 33.55 ± 0.08 | 27.13 ± 1.69a | 18.60 ± 1.59b | **t**(**4**)**= -6.82**  ***p < 0.001*** | 24.82 ± 0.05 | 25.93 ± 1.12a | 20.07 ± 1.42b | **t**(**4**)**= -5.94**  ***p < 0.001*** |
| 18 | (*E)-C18*:1n7 | 1.56 ± 0.02 | 0.99 ± 0.13a | 1.76 ± 0.17b | **t**(**4**)**= 6.27**  ***p < 0.001*** | 1.27 ± 0.02 | 0.82 ± 0.09a | 2.44 ± 0.24b | **t**(**4**)**= 10.97**  ***p < 0.001*** |
| 19 | C18:2n6D | 0.00 ± 0.00 | 1.29 ± 0.22a | 0.00 ± 0.00b | **t**(**4**)**= -10.30**  ***p < 0.001*** | 0.00 ± 0.00 | 0.00 ± 0.00 | 0.00 ± 0.00 | **/** |
| 20 | (*Z, Z*)-C18:2n6 | 26.09 ± 0.72 | 12.85 ± 0.94a | 11.21 ± 3.27a | **t**(**4**)**= -0.83**  **p = 0.479** | 19.85 ± 0.08 | 12.79 ± 1.08a | 8.32 ± 1.57b | **t**(**4**)**= -4.98**  ***p = 0.011*** |
| / | C18:2n6D + (*Z, Z*)-C18:2n6 | 26.09 ± 0.72 | 14.14 ± 0.77a | 11.21 ± 3.27a | **t**(**4**)**= -1.51**  **p = 0.257** | 19.85 ± 0.08 | 13.79 ± 1.08a | 8.32 ± 1.57b | **t**(**4**)**= -4.98**  ***p = 0.011*** |
| 21 | C18:3n6 | 0.00 ± 0.00 | 0.00 ± 0.00a | 0.43 ± 0.12b | **t**(**4**)**= 6.12**  ***p = 0.025*** | 0.00 ± 0.00 | 0.00 ± 0.00a | 0.38 ± 0.03b | **t**(**4**)**= 21.40**  ***p < 0.001*** |
| 22 | C18:3n3 | 0.96 ± 0.03 | 0.51 ± 0.03a | 0.59 ± 0.14a | **t**(**4**)**= 0.89**  **p = 0.455** | 35.54 ± 0.23 | 13.30 ± 1.83a | 5.69 ± 1.39b | **t**(**4**)**= -5.73**  ***p = 0.005*** |
| 23 | CLA 1 | 0.71 ± 0.06 | 6.78 ± 1.83 | 4.11 ± 0.19 | **t**(**4**)**= -2.51**  ***p = 0.125*** | 0.00 ± 0.00 | 3.67 ± 0.65 | 5.17 ± 0.97 | **t**(**4**)**= 2.19**  **p = 0.102** |
| 24 | CLA 2 | 1.08 ± 0.60 | 1.47 ± 0.52 | 1.28 ± 0.47 | **t**(**4**)**= -0.48**  **p = 0.653** | 0.00 ± 0.00 | 3.67 ± 0.51 | 5.23 ± 1.03 | **t**(**4**)**= 2.36**  **p = 0.101** |
|  | Bold font indicates statistical significance. Means followed by letters in common are not significantly different. D = deuterated; CF = 100% Chicken feed; BSF-CF = Black soldier fly produce on CF; BSF-CFD= BSF-CF with deuterated water. FL = 40% chicken feed + 60% flax cake; BSF-FL = Black soldier fly produce on FL; BSF-FLD= BSF-FL with deuterated water. Letter Z and E describe the absolute stereochemistry of double bonds in the molecule | | | | | | | | |

| 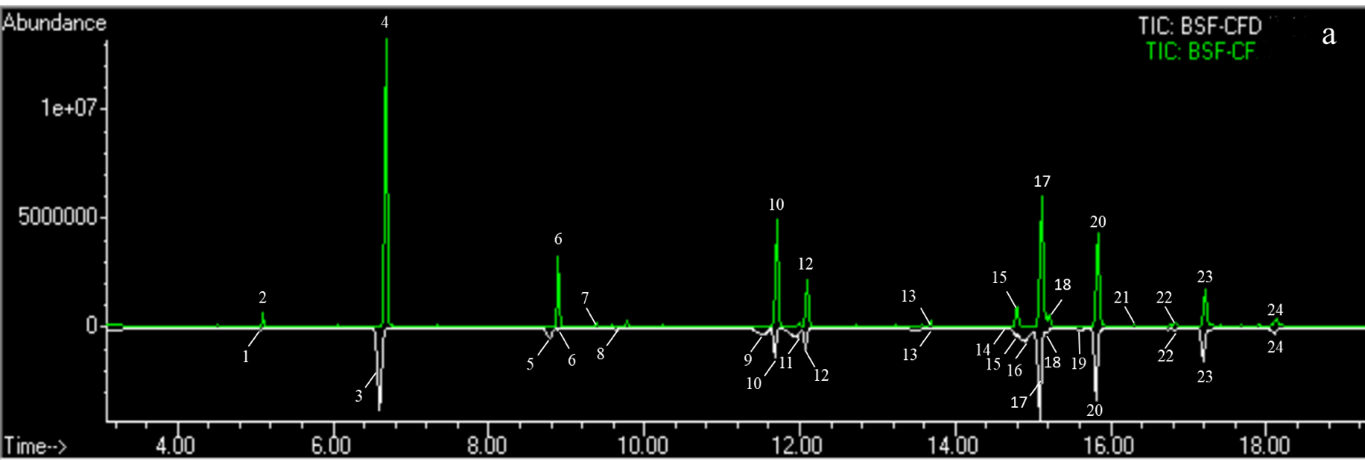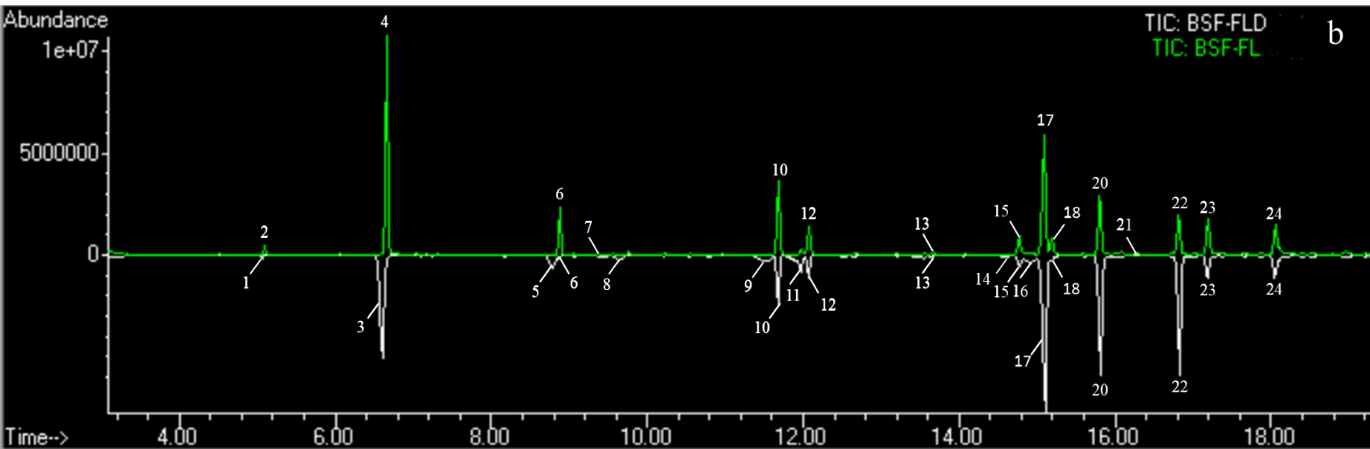 |
| --- |
| Figure S1 Gas chromatography - mass spectrometry total ion chromatograms (TIC) of fatty acid methyl-esters of black soldier fly (BSF) prepupae  (a) TIC of BSF prepupae produced on chicken feed (100%) with tap water (BSF-CF; in green) and with deuterated water (BSF-CFD; in white).  (b) TIC of BSF prepupae produced on a mixture of chicken feed (40%) and flax cake (60%) with tap water (BSF-FL; in green) and with deuterated water (BSF-FLD; in white).  The numbers correspond to the fatty acid identification (ID number Tables S1) |

| a  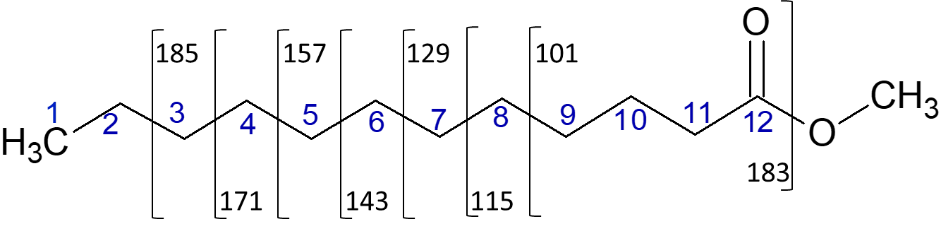  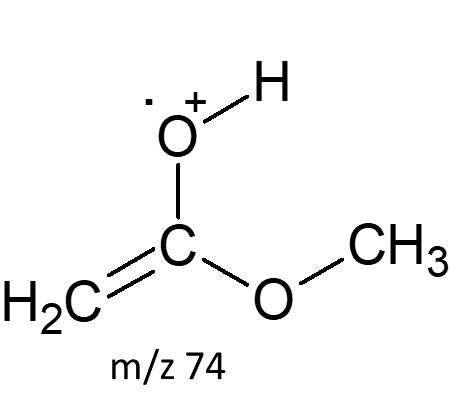 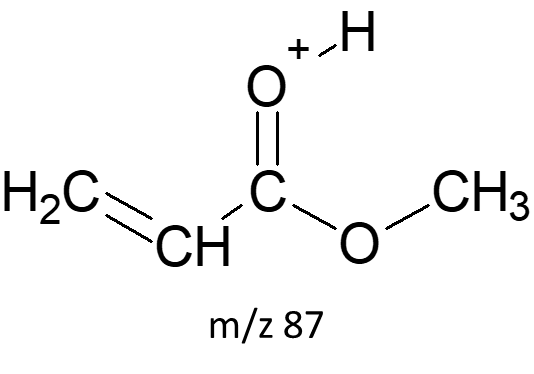 |
| --- |
| b   |
| Figure S2 Structure of lauric acid methyl ester and its major deuterated species from black soldier fly  (a) Lauric acid methyl ester structure and fragmentation  (b) Structure of major deuterated lauric acid methyl ester species |
